# Draft Genome Sequence of *Lysobacter capsici* AZ78, a Bacterium Antagonistic to Plant-Pathogenic Oomycetes

**DOI:** 10.1128/genomeA.00325-14

**Published:** 2014-04-24

**Authors:** Gerardo Puopolo, Paolo Sonego, Kristof Engelen, Ilaria Pertot

**Affiliations:** a Department of Sustainable Agro-Ecosystems and Bioresources, Research and Innovation Centre, Fondazione Edmund Mach (FEM), San Michele all'Adige, Italy; b Department of Computational Biology, Research and Innovation Centre, Fondazione Edmund Mach (FEM), San Michele all'Adige, Italy

## Abstract

*Lysobacter capsici* AZ78, isolated from tobacco rhizosphere, effectively controls *Phytophthora infestans* and *Plasmopara viticola* on tomato and grapevine plants, respectively. We report the first draft genome sequence of the *L. capsici* species.

## GENOME ANNOUNCEMENT

The bacterial genus *Lysobacter* (1) represents a source of biocontrol agents capable of protecting plants against diseases caused by pathogenic microorganisms (2). The species *Lysobacter capsici* encompasses a few bacterial strains that effectively control different plant-pathogenic bacteria, fungi, nematodes, and oomycetes (3–6). Recently, we demonstrated that applications of *L. capsici* AZ78 can control *Phytophthora infestans* and *Plasmopara viticola*, which are two remarkable plant-pathogenic oomycetes of tomatoes and grapevines, respectively (7; G. Puopolo, A. Cimmino, M. C. Palmieri, O. Giovannini, A. Evidente, and I. Pertot, submitted for publication). Because of its importance as a potential biocontrol agent, we analyzed the draft genome sequence of *L. capsici* AZ78.

The genome of *L. capsici* AZ78 was sequenced by using the Illumina GAIIx system. A total of 7,512,266 filtered reads for *L. capsici* AZ78 were assembled into 142 contigs (*N*_50_ length, 139,986 bp), with an average coverage of 40.0×, using the A5 pipeline (8). The genome consists of 6,315,650 bases with 102 contigs of > 1,000 bp each and a G+C content of 66.43%, which is similar to the content of the type strain of the *L. capsici* species (65.4% [3]). The genome annotations were performed by the NCBI Prokaryotic Genomes Annotation Pipeline utilizing GeneMarkS (9). Automated annotation was performed using the RAST annotation server (10). The *L. capsici* AZ78 genome contains 5,448 predicted coding sequences. Most of the coding sequences (3,654) do not belong to the RAST subsystems, while of the remaining sequences, 1,794 were assigned functions and 93 were considered to encode hypothetical proteins. The number of genes coding for RNAs was determined by using the software Barnap implemented in Prokka (11). The *L. capsici* AZ78 genome contains 93 predicted RNAs, of which 1 is a transfer-messenger RNA (tmRNA), 7 are rRNAs, and 85 are tRNAs.

The RAST analysis brought out the presence of genes coding for resistance to drugs and heavy metals. Additionally, this analysis showed that the *L. capsici* AZ78 genome contains genes involved in copper ion transport, homeostasis, uptake, and resistance, a property that made it possible to combine this biological control agent with copper-based fungicides (7).

As expected, the *L. capsici* AZ78 genome contains a high number of genes coding for lytic enzymes (2). Specifically, the lytic weaponry of *L. capsici* AZ78 encompasses chitinases, glucanases, lipases, xylanases, and several enzymes with proteolytic activity.

The availability of the draft genome of *L. capsici* AZ78 will help elucidate the mechanism of action of this bacterial strain against plant-pathogenic oomycetes and will give background knowledge that is useful for the registration of *L. capsici* AZ78 as an active ingredient in plant protection products.

### Nucleotide sequence accession numbers.

This whole-genome shotgun project has been deposited at DDBJ/EMBL/GenBank under the accession no. JAJA00000000. The version described in this paper is version JAJA01000000.
